# Hospital Floors

**Published:** 1902-07-12

**Authors:** 


### EDITOR'S LETTER-BOX.

[Our Correspondents are reminded that prolixity is a great bar to publication, and that brevity of style and conciseness of statement greatly facilitate early insertion.]

### HOSPITAL FLOORS.

"HOSPITAL SECRETARY" writes: It has been suggested to my committee that they should obtain your opinion as to the desirability of having flooring boards impregnated with paraffin. Some remarks upon the subject are made in Sir Henry Burdett's book on hospital construction, and the committee will consider it a great favour if you will let me know what you consider to be the best method for hospital ward flooring in the light of recent experiences. We propose to use Austrian oak, kiln-dried, 1¼ inch, laid in mastic on cement.

[We are of opinion that the best form of hospital floor available at the present time, if we may judge by results, is undoubtedly well-seasoned teak of the best quality, properly laid under the most approved method, care being taken if tongued boarding be used that the workmanship is of the best, and uniformly so throughout. We inspected the other day some teak floors after four years' wear in a great naval hospital, where they object to tonguing because they had found it unsatisfactory, and had consequently laid the floors like a ship's deck, and caulked them. We never saw a more satisfactory result than was here secured, and the medical staff were high in praise of this method so far as their experience went. We have had all kinds of new material for floors under inspection and trial during recent years, but we are not yet satisfied that the ideal flooring has been found. We are at the moment making an experiment with a new material in a small ward, but cannot at present say what the results are likely to be. The opinion here given is based upon the continuous experience of thirty years of fairly close observation and experiments, and in our opinion there is nothing available which in all respects compares with teak for the purposes of ward floors. That is why we continue to recommend it, but this recommendation is qualified by the most earnest insistence that unless the teak be thoroughly seasoned, and the workmanship and plan adopted for laying the floors are of the very best that can be obtained, the result cannot be guaranteed to prove satisfactory.—ED. THE HOSPITAL.]

